# Factors influencing the health satisfaction of users of public health and medical institutions in South Korea

**DOI:** 10.3389/fpubh.2022.1079347

**Published:** 2023-01-16

**Authors:** Kichan Yoon, Munjae Lee

**Affiliations:** ^1^The Institute for Democracy, Seoul, Republic of Korea; ^2^Department of Medical Humanities and Social Medicine, Ajou University School of Medicine, Suwon, Republic of Korea; ^3^Medical Research Collaborating Center, Ajou Research Institute for Innovative Medicine, Ajou University Medical Center, Suwon, Republic of Korea

**Keywords:** subjective quality of life, health satisfaction, public health institutions, health promotion project, EQ-5D-5L, South Korea

## Abstract

**Introduction:**

In this study, we investigated the health satisfaction levels of users of regional health and medical institutions in South Korea and the influencing factors.

**Methods:**

We included randomly selected 300 people with experience in using health and medical institutions from panel data targeting the entire nation. We used questionnaire (EQ-5D-5L) and sociodemographic characteristics to analyze the health satisfaction. EQ-5D-5L was used to measure health-related quality of life in five areas: mobility, self-management, daily activities, pain/discomfort, and anxiety/depression. Hierarchical linear regression analysis was performed in three steps to examine the factors influencing health satisfaction.

**Results:**

The analysis showed that the health satisfaction was positively (+) correlated with monthly income, mobility, self-management, daily life, pain/discomfort, and anxiety/depression, and negatively (−) correlated with the number of chronic diseases and type of health insurance. The influencing factors in Step 1 and 2 were chronic diseases (β = −0.380, −0.385), respectively. The influencing factors in Step 3 were pain/discomfort (β = 0.202), anxiety/depression (β = 0.257), and the number of chronic diseases (β = −0.222).

**Discussions:**

The current data suggested that regional health and medical institutions should focus their services on residents with chronic diseases. Moreover, they should expand physical activities to relieve physical pain or discomfort and provide services related to mental health. To accomplish these, we suggested that the government will need to promote post-service health checkup results as a key project, provide user-customized services, provide online services utilizing ICT, expanding the government's financial support, and building infrastructure.

## 1. Introduction

In relation to COVID-19, South Korea has demonstrated a consistently effective response through appropriate measures at the national level (1). In this effective response, the role of regional health and medical institutions centered on public health centers is significant. According to Korea's Regional Public Health Act and the Act on Special Measures for Health and Medical Care in Rural Areas, regional health and medical institutions have their respective duties and functions. They also include different types, including public health centers, health and medical centers, branch offices of health centers, and health life supporting centers, established to provide health and medical services for vulnerable areas of health care (2, 3). Public health centers and health and medical centers can be established by local government ordinances, whereas health care centers can be set up with the approval of the Ministry of Health and Welfare (4, 5).

Health-promotion projects for local residents are centered on city, county, and *gu* (x) public health centers, and the planning, execution, and evaluation units of such projects are consistent with those of local government health centers. They are differentiated from basic. local self-governments in that public health centers perform the primary treatment (at the clinic level) and that health and medical centers conduct secondary treatment (at the hospital level); both public health centers and health and medical centers are collectively defined as public health centers (6–9). Therefore, regional health and medical institutions promote various projects to meet the diverse needs of local residents and raise their health levels (10–13). Through the implementation of these projects, the health-related quality of life of local residents is improved, thereby increasing their health satisfaction (14–17).

As of 2019, South Korea has 256 public health centers, with the same number of autonomous districts in the Special City and Metropolitan Cities, and one public health center operating in Sejong Metropolitan Autonomous City (18). In addition, 1,417 branch offices of health centers and 1,894 health care centers are in operation. Public health centers are established by local governments mainly to promote the health of local residents and to manage and prevent diseases, whereas the branch offices of health centers should be located in towns and villages. Health and medical centers are public health centers equipped with hospital facilities, in accordance with the Medical Act (19). Health care centers, established in vulnerable areas of health care, contribute to the health promotion of local residents and are mainly installed in rural areas. Among the public health centers subject to the Regional Public Health Act, health life support centers are established to prevent chronic diseases and support the healthy lifestyles of local residents (20).

Regarding personnel status, regional health and medical institutions have 8,829 employees as of 2019, with a largely equal composition ratio of public and non-public officials (e.g., contractors, nonregular workers, outsourced workers, and part-time workers). Regarding their usage status, the number of residents participating in health-promotion projects as of 2019 was about four times higher, in terms of total users, than that of health care users. In terms of the actual users with duplicates removed, the number was 2,186,026, corresponding to 2.9 times that of 6,394,510 users of health services (21). Accordingly, regional health and medical institutions make a significant contribution to the health promotion of local residents in rural areas where health care infrastructure is relatively lacking.

Regional health and medical institutions conduct projects by dividing them into integrated and other health-promotion projects. To improve the health level of residents and enhance health equity, institutions are implementing 13 types of projects: smoking prohibition, sobriety, physical activity, nutrition, preventive management of obesity, oral health, preventive management of cardiovascular and cerebrovascular diseases, oriental medicine health promotion, preventive management of atopy and asthma, maternal and child health, dementia management, community-based rehabilitation, and visiting health care (22). Other health-promotion projects include nine programs: mental health project management, health project management for older adults, health examination result counseling, denture (false teeth) provision, tuberculosis management, Hansen's disease management, sexually transmitted infection disease management, maternal and child health programs, and vaccination campaigns (23).

Although residents' participation in 22 health-promotion projects is actively encouraged and centered on the local community, research on the influencing factors affecting the health level of the users of regional health and medical institutions is insufficient. As mentioned above, health-related quality of life influences health satisfaction with public health and medical institution projects. Therefore, we intended to investigate the health satisfaction levels of users of regional health and medical institutions in South Korea through various health promotion projects and the health-related quality of life factors affecting health satisfaction. For this study, panel data targeting the entire nation were used.

This study has several differences from the conventional studies that have analyzed the effect on the health satisfaction of patients who use medical services in the hospitals. First, the subjects of this study are not the patients who are using hospitals, but the residents using public health centers, which are local medical institutions led by the government. Second, regional health and medical institutions are the place where projects to improve the health level of local residents are mainly implemented rather than medical services caused by diseases. Third, since they are nation-leading public institutions, they do not pursue profits–unlike private hospitals or clinics–and mainly deal with national health problems, such as pandemics. Therefore, this study focuses on analyzing how nation-leading health projects affect the health satisfaction of local residents who use regional health and medical institutions. In particular, Korea's health insurance system which has the form of NHI (National Health Insurance) uses private hospitals or clinics, but apart from this, it has a distinct characteristic that there are various health projects implemented by the nation for the health of local residents.

## 2. Methodology

The subject of this study is public health centers, which are Public Health and Medical Institutions belonging to local governments. This is because the post-service service of health checkup results is a project implemented by public health centers. To analyze the health satisfaction of the users of regional health and medical institutions and the influencing factors, we used health satisfaction as a dependent variable, and standardized 5-level EuroQol 5-dimensional questionnaire (EQ-5D-5L), a tool for measuring the quality of life affecting health satisfaction, as an influencing factor. The EQ-5D-5L was developed to improve the sensitivity and ceiling effect of the existing EQ-5D-3L (24), where the five areas are the same but the measurement level has been expanded from the three-level to the five-level Likert scale. As shown in Table 1, the influencing factors consisted of five areas: mobility, self-management, daily activities, pain/discomfort, and anxiety/depression. We then conducted hierarchical linear regression analysis on demographic and quality-of-life variables to measure the magnitude of the influencing variables and their influence.

**Table 1:** Questionnaire used to measure subjective quality of life.

**Item**	**Before use**					**After use**				
I have no problem walking (Mobility)	➀	➁	➂	➃	➄	➀	➁	➂	➃	➄
I have no problem washing or dressing by myself (Self-management)	➀	➁	➂	➃	➄	➀	➁	➂	➃	➄
I have no problems with my daily life (Daily activities)	➀	➁	➂	➃	➄	➀	➁	➂	➃	➄
I have no pain or discomfort (Pain/Discomfort)	➀	➁	➂	➃	➄	➀	➁	➂	➃	➄
I am not anxious or depressed (Anxiety/Depression)	➀	➁	➂	➃	➄	➀	➁	➂	➃	➄

## 3. Results

### 3.1. Demographic characteristics

To analyze the influencing factors on health satisfaction targeting the users of regional health and medical institutions, we used a 14-item questionnaire composed of the following: one question on health satisfaction, five questions on health-related quality of life (EQ-5D-5L), and eight questions on demographic characteristics. For the survey, a specialized agency was commissioned to conduct from November 12 to 18, 2020. In terms of the survey subjects, 300 residents who had experience of using public health centers and 200 residents who did not have experience of using public health centers were randomly assigned among the panels held by the institution. In other words, the survey subjects were extracted based on the experience of using public health centers. Next, 150 people were assigned for each, according to whether or not they have experience of using health checkup post-services among the local residents who have experience of using public health centers. The survey was conducted until 300 people were assigned among the panels held by the survey agency. The questionnaire was written in Korean, and among the questionnaire items, negative sentences were reverse-coded into positive ones. For hierarchical regression analysis, PASW Statistics 18 version was utilized. Table 2 gives the demographic characteristics of the sample.

**Table 2:** Sociodemographic characteristics of study participants (unit: Person, %).

**Variable**	**Item**	**Frequency**	**Ratio**
Use experience of health institution	Any	300	40.6
	None	200	59.4
Service usage experience	Any	150	50.0
	None	150	50.0
Number of chronic diseases	0	175	35.0
	1	194	38.8
	2 or more	131	26.2
Gender	Male	242	48.4
	Female	258	51.6
Age	40 s	233	58.6
	50 s	153	30.6
	60 s or older	54	10.8
Marriage	Married	407	81.7
	Single	91	18.3
Health insurance type	Workplace	335	67.0
	Region	147	29.2
	Other	19	3.8
Monthly income	< 2,000 USD	42	8.4
	< 4,000 USD	169	33.8
	< 6,000 USD	166	33.2
	6,000 USD or more	123	24.6
Number of family members	None	10	2.0
	1	49	9.8
	2	86	17.2
	3 or more	355	71.0
Residential area	Major city	203	40.6
	Rural areas	297	59.4

### 3.2. Factors influencing health satisfaction

We analyzed the factors affecting the health satisfaction of local residents who had experience using regional health and medical institutions. In conducting hierarchical linear regression analysis, we verified the causal relations between health satisfaction and the number of chronic diseases in Step 1, between chronic diseases and sociodemographic characteristics in Step 2, and between chronic diseases, sociodemographic characteristics, and health-related quality of life indicators in Step 3. We checked for meaningful changes in explanatory power for each step. The results are shown in Table 3.

**Table 3:** Correlation analysis.

	**Health satisfaction**	**Number of diseases**	**Sex**	**Region**	**Civil status**	**Monthly income**	**Number of household members**	**Age**	**Health insurance**	**Mobility**	**Self-management**	**Daily life**	**Pain/** **Discomfort**	**Anxiety/** **Depression**
Health satisfaction	1.000	−0.380^*^	0.008	−0.045	−0.040	0.071	0.048	−0.081	−0.099^*^	0.399^*^	0.307^*^	0.406^*^	0.468^*^	0.463^*^
Number of diseases	−0.380^*^	1.000	−0.023	−0.030	−0.079^*^	0.049	0.035	0.202^*^	0.045	−0.252^*^	−0.228^*^	−0.311^*^	−0.310^*^	−0.225^*^
Sex	0.008	−0.023	1.000	0.021	0.007	−0.088^*^	−0.019	−0.003	0.158^*^	−0.028	0.010	−0.002	−0.094^*^	−0.042
Region	−0.045	−0.030	0.021	1.000	−0.079^*^	−0.070^**^	−0.052	0.033	0.071	−0.007	−0.027	−0.071^**^	−0.038	0.001
Civil status	−0.040	−0.079^*^	0.007	−0.079^*^	1.000	−0.262^*^	−0.352^*^	−0.245^*^	0.197^*^	−0.003	0.003	0.037	0.043	−0.074^**^
Monthly income	0.071^**^	0.049	−0.088^*^	−0.070^**^	−0.262^*^	1.000	0.327^*^	−0.001	−0.380^*^	0.054	0.032	0.021	0.060^**^	0.030
Number of household members	0.048	0.035	−0.019	−0.052	−0.352^*^	0.327^*^	1.000	−0.074^**^	−0.149^*^	0.004	0.009	0.021	0.058	0.036
Age	−0.081	0.202^*^	−0.003	0.033	−0.245^*^	−0.001	−0.074^*^	1.000	0.154^*^	−0.116^*^	−0.116^*^	−0.140^*^	−0.073^**^	0.012
Health insurance	−0.099^*^	0.045	0.158^*^	0.071^**^	0.197^*^	−0.380^*^	−0.149^*^	0.154^*^	1.000	−0.106^*^	−0.106^*^	−0.056	−0.081^*^	−0.086^*^
Mobility	0.399^*^	−0.252^*^	−0.028	−0.007	−0.003	0.054	0.004	−0.116^*^	−0.106^*^	1.000	0.730^*^	0.689^*^	0.540^*^	0.429^*^
Self-management	0.307^*^	−0.228^*^	0.010	−0.027	0.003	0.032	0.009	−0.116^*^	−0.106^*^	0.730^*^	1.000	0.716^*^	0.434^*^	0.322^*^
Daily life	0.406^*^	−0.311^*^	−0.002	−0.071^**^	0.037	0.021	0.021	−0.140^*^	−0.056	0.689^*^	0.716^*^	1.000	0.564^*^	0.399^*^
Pain/ Discomfort	0.468^*^	−0.310^*^	−0.094^*^	−0.038	0.043	0.060^**^	0.058	−0.073^**^	−0.081^*^	0.540^*^	0.434^*^	0.564^*^	1.000	0.459^*^
Anxiety/ Depression	0.463^*^	−0.225^*^	−0.042	0.001	−0.074	0.030	0.036	0.012	−0.086^*^	0.429^*^	0.322^*^	0.399^*^	0.459^*^	1.000

* P < 0.05,

** P < 0.01.

We analyzed the correlations among the health satisfaction and number of chronic diseases as dependent variables, the variables for socio-demographic characteristics, and the variables for health-related quality of life. The variables positively (+) correlated with health satisfaction as a dependent variable were monthly income, mobility, self-management, daily life, pain/discomfort, and anxiety/depression. Meanwhile, among the variables showing a negative (-) correlation with health satisfaction as a dependent variable, the number of chronic diseases and type of health insurance had a statistically significant relation.

Specifically, among the variables indicating sociodemographic characteristics other than health-related variables, monthly income and health satisfaction showed a positive correlation, whereas regional insurance showed a negative correlation. Therefore, the post-service from the results of health checkups by local health institutions should intensively manage those who have low-income levels and have taken up local health insurance.

Next, hierarchical linear regression analysis was performed in three steps to examine the factors influencing health satisfaction. As a result of hierarchical regression analysis, there was no statistically significant change in the regression coefficient between Step 1 and 2 (R^2^ = 0.146), while the regression coefficient was statistically significant between Step 2 and 3 (R^2^ = 0.347). This means that the health-related quality of life (EQ-5D-5L) has a significant impact on user satisfaction. The value of the Durbin–Watson verification for multicollinearity was 1.948—close to 2.0—indicating the absence of multicollinearity. The results are shown in Table 4.

**Table 4:** Hierarchical multiple regression model summary.

**Model**	**R square**	**Adjusted R square**	**Std. error of the estimate**	**Statistic change**					**Durbin-Watson**
				**R squared change**	**F square**	**df1**	**df2**	**Sig. F change**	
1	0.144	0.142	0.64423	0.144	79.070	1	469	0.000	
2	0.160	0.146	0.64304	0.016	1.248	7	462	0.275	
3	0.365	0.347	0.56212	0.205	29.519	5	457	0.000	1.948

In Step 1, we investigated the influence of the number of chronic diseases on health satisfaction. As shown in Table 5, the F-value of the model in Step 1 was 79.079, which was suitable as a regression model with a 95% confidence level. In addition, the regression coefficient (R^2^), indicating explanatory power, was 0.142, which was found to explain 14.2% of the entire regression model.

**Table 5:** Factors influencing health satisfaction (Model 1).

**Independent variable**	**Model 1**		
	**SE**	β **(Standardized)**	***t*** **-value**
(Constant)	0.079		48.208
Number of diseases	0.038	−0.380	−8.892^*^
Statistic	R^2^ = 0.142, F = 79.079^*^		

* P < 0.05.

The hierarchical linear regression analysis in Step 1 showed that the number of chronic diseases had a negative effect on health satisfaction (β = −0.380). In other words, the higher the number of chronic diseases, the lower the health satisfaction. Thus, chronic diseases are the main cause of the low health satisfaction of local residents. Accordingly, regional health and medical institutions should operate post-services from health examination results centered on chronic diseases.

For the hierarchical linear regression model in Step 2, we included sex, region, marriage, monthly income, number of households, age, and health insurance type, which indicate socio-demographic characteristics. The F-value representing model fitness was 11.013 (Table 6), which was statistically significant, whereas the explanatory power was 14.6%, which showed no significant change from that in the first step. The analysis revealed that the number of chronic diseases (−0.385) had a negative effect on health satisfaction, as in Step 1. However, variables representing sociodemographic characteristics did not have a significant effect on health satisfaction.

**Table 6:** Factors influencing health satisfaction (Model 2).

**Independent variable**	**Model 1**			**Model 2**		
	**SE**	β **(Standardized)**	***t*** **-value**	**SE**	β **(Standardized)**	***t*** **-value**
(Constant)	0.079		48.208	0.304		12.928
Number of diseases	0.038	−0.380	−8.892^*^	0.039	−0.385	−8.809^*^
Sex				0.060	0.013	0.300
Region				0.061	−0.053	−1.219
Civil status				0.093	−0.046	−0.933
Monthly income				0.038	0.052	1.057
Number of family members				0.051	0.019	0.387
Age				0.048	−0.004	−0.088
Health insurance				0.073	−0.048	−0.993
Statistic	R^2^ = 0.146, F = 11.013^*^					

* P < 0.05.

Finally, to complete the three-step hierarchical linear regression analysis of factors affecting health satisfaction, we added the health-related quality-of-life variables. As shown in Table 7, the F-value representing model fitness was 20.222, suggesting suitability at the 95% significance level. In particular, the regression coefficient (R2) to explain the regression model was 34.7%, showing a statistically significant change in explanatory power compared with Steps 1 and 2.

**Table 7:** Factors influencing health satisfaction (Model 3).

**Independent variable**	**Model 1**			**Model 2**			**Model 3**		
	**SE**	β **(Standardized)**	***t*** **-value**	**SE**	β **(Standardized)**	***t*** **-value**	**SE**	β **(Standardized)**	***t*** **-value**
(Constant)	0.079		48.208	0.304		12.928	0.328		6.400
Number of diseases	0.038	−0.380	−8.892^*^	0.039	−0.385	−8.809	0.037	−0.222	−5.434^*^
Sex				0.060	0.013	0.300	0.053	0.044	1.154
Region				0.061	−0.053	−1.219	0.054	−0.039	−1.022
Civil status				0.093	−0.046	−0.933	0.082	−0.042	−0.974
Monthly income				0.038	0.052	1.057	0.033	0.040	0.931
Number of household members				0.051	0.019	0.387	0.045	0.000	0.001
Age				0.048	−0.004	−0.088	0.042	−0.013	−0.327
Health insurance				0.073	−0.048	−0.993	0.064	−0.020	−0.465
Mobility							0.047	0.099	1.612
Self-management							0.051	−0.054	−0.894
Daily life							0.051	0.086	1.409
Pain/Discomfort							0.032	0.202	4.065^*^
Anxiety/Depression							0.028	0.257	5.862^*^
Statistic	R^2^ = 347^*^, F = 20.222^*^								

* P < 0.05.

The influencing factors in Step 3 were pain/discomfort (β = 0.202), anxiety/depression (β = 0.257), and the number of chronic diseases (-0.222). Thus, the number of chronic diseases again showed a negative effect on health satisfaction, but a reduction in pain/discomfort or anxiety/depression had a positive effect on health satisfaction. Accordingly, to activate post-services from health checkup results, regional health and medical institutions in Korea should formulate an exercise prescription program that can reduce pain or discomfort of local residents or a mental health program that can reduce anxiety and depression.

## 4. Discussion and conclusion

First of all, the correlation between health satisfaction and independent variables was analyzed. Variables that have a positive (+) correlation with health satisfaction include monthly income, mobility, self-management, daily life, pain/discomfort, and anxiety/depression, whereas variables that have a negative (-) correlation were statistically significant with the number of chronic diseases and the subscription type of health insurance. In other words, users with high income and high health-related quality of life have a positive relationship with health satisfaction, while users who have many chronic diseases or who take out a local insurance policy have a negative relationship with health satisfaction. Those who are reduced in mobility or who are relatively self-employed have a negative correlation with health satisfaction.

Next, We conducted hierarchical linear regression analysis of the factors influencing the health satisfaction of local residents using regional health and medical institutions in Korea. The results showed that the higher the number of chronic diseases, the more negatively affected their health satisfaction. We also found that health-related quality of life variables, particularly pain/discomfort or anxiety/depression, had a positive effect on health satisfaction. Thus, regional health and medical institutions should focus their services on residents with chronic diseases. Moreover, they should expand physical activities to relieve physical pain or discomfort and provide services related to mental health, such as anxiety and depression. Based on these results, we formulated the following policy implications.

First, post-service health checkup results, the effects of which on the health of local residents were verified in our study, should be promoted as a key project for local residents. In particular, the project should aim to improve the health level of the entire local community through resource sharing *via* public–private cooperation.

Second, given that the number of chronic diseases affects health satisfaction, regional health and medical institutions in Korea should provide user-customized services according to the changes in chronic diseases by life cycle. It is important to develop projects that reflect the characteristics of individuals, such as age, sex, and household properties.

Third, considering the important role of regional health and medical institutions in Korea's rural areas where health care infrastructure is lacking, so-called untact services utilizing Information and Communications Technology (ICT) should be expanded. Such an expansion should account for the regional nature of the large older adult population. In particular, services related to mental health and chronic diseases should be provided using telemedicine or ICT-based untact services.

Fourth, improvements to the systemic problem should be implemented. As present, residents are compelled to use only local health and medical institutions in their current place of resident registration place. Local residents who need to use them should be allowed to utilize all regional health and medical institutions regardless of their residence. Particularly, it is necessary to increase accessibility by allowing them to use regional health and medical institutions close to their places of residence.

Lastly, for regional health and medical institutions to raise the level of health satisfaction of local residents, it is necessary to not only expand the government's financial support but also build an infrastructure that enables the provision of integrated services. It is necessary to provide data-based health services by establishing an integrated health-related platform based on big data. Because there is a positive correlation between user mobility and health satisfaction, it is important to use a public health center close to the residence.

In conclusion, this study found that the number of chronic diseases have an impact on the satisfaction of users of public health centers operated by local governments and that pain/discomfort or anxiety/depression among the quality-of-life indicators were influential in that. In some studies, the influencing factors on patient satisfaction include physical structure, interaction between patients and healthcare workers, administrative and medical services, quality of nursing services, etc., (25). However, as a local medical institution in Korea, public health centers are differentiated in that they improve the health-related quality of life by performing projects that can be resolved at the community level, such as chronic disease management, mental health management, etc., rather than dealing with diseases or surgery as a public institution.

## Data availability statement

The datasets presented in this article are not readily available because we cannot publicly make them available due to the data provider's regulations. Requests to access the datasets should be directed to KY; ykichan08@gmail.com.

## Ethics statement

Ethical review and approval was not required for the study on human participants in accordance with the local legislation and institutional requirements. The patients/participants provided their written informed consent to participate in this study.

## Author contributions

KY collected the data, processed statistical data, and drafted the manuscript. ML contributed to the study design, manuscript edits, and supervised the project. Both authors read, contributed to the research design, and approved the final manuscript.

## References

[1] Park Y Park S Lee M. Digital health care industry ecosystem: network analysis. J Med Internet Res. (2022) 24:e37622. 10.2196/37622 35976690 PMC9434390

[2] Byrne A Barber R Lim CH. Impact of the COVID-19 pandemic–a mental health service perspective. Prog Neurol Psychiatry. (2021) 25:27–33b. 10.1002/pnp.708

[3] Park SH Lee MS Oh YJHP. Management direction reorganization of public health center functions through analysis of medical service status by public health care institutions. Health Policy Manag. (2022) 32:3–13. 10.4332/KJHPA.2022.32.1.3

[4] Na BJ. How impact the pandemic of COVID-19 to the restructuring of public health centers in the future. Public Health Aff . (2021) 5. 10.29339/pha.21.5

[5] Seong H Hyun HJ Yun JG Noh JY Cheong HJ Kim WJ. Comparison of the second and third waves of the COVID-19 pandemic in South Korea: importance of early public health intervention. Int J Infect Dis. (2021) 104:742–5. 10.1016/j.ijid.2021.02.004 33556610 PMC7863747

[6] de Deus DR Parreira FR Paiva MdFC de Lourdes Miguel Alcântara M de Cesaro Antunes P Batista SM. TEIA: intersectoral topics and strategies with Community Health Workers (CHWs), education, communication, and health promotion in times of pandemic. In: International Handbook of Teaching and Learning in Health Promotion. Cham: Springer. (2022). p. 167–87. 10.1007/978-3-030-96005-6_12

[7] Kegler MC Rigler J Honeycutt S. The role of community context in planning and implementing community-based health promotion projects. Eval Program Plan. (2011) 34:246–53. 10.1016/j.evalprogplan.2011.03.004 21555048

[8] Kim D. Health plan 2030 for the promotion of national health. In: Korea Institute for Health and Social Affairs Health Welfare Issue & Focus, Vol. 79. (2021). p. 1–6.

[9] Till M Abu-Omar K Ferschl S Abel T Pfeifer K Gelius PJE. Implementing the capability approach in health promotion projects: recommendations for implementation based on empirical evidence. Eval Program Plan. (2022) 95:102149. 10.1016/j.evalprogplan.2022.102149 36029567

[10] Malcarney MB Pittman P Quigley L Horton K Seiler NJHsr. The changing roles of community health workers. Health Serv Res. (2017) 52:360–82. 10.1111/1475-6773.12657 28127766 PMC5269544

[11] Pakhnenko OM Kulibaba V Palienko M. Health care provision in state institutions in the context Of COVID-19. Health Econ Manag Rev. (2022) 3:17–25. 10.21272/hem.2022.2-02

[12] RYU J-A. The effect of distribution of regional health care institutions on subjective wellbeing of the elderly in seoul: a multi-level analysis considering the moderating effect of subjective economic status. J Korea Gerontol Soc. (2022) 42:691–712. 10.31888/JKGS.2022.42.3.691

[13] Yessimov N Izmailova N Yessimov DJ. Integration of primary healthcare and public health. Int J Electron Healthc. (2021) 11:289–306. 10.1504/IJEH.2021.117826

[14] Kaplan RM Hays RD. Health-related quality of life measurement in public health. Ann Rev Public Health. (2022) 43:355–73. 10.1146/annurev-publhealth-052120-012811 34882431

[15] Kim ES Delaney SW Tay L Chen Y Diener E Vanderweele TJ. Life satisfaction and subsequent physical, behavioral, and psychosocial health in older adults. Milbank Q. (2021) 99:209–39. 10.1111/1468-0009.12497 33528047 PMC7984669

[16] Nakamura JS Delaney SW Diener E VanderWeele TJ Kim ES. Are all domains of life satisfaction equal? Differential associations with health and well-being in older adults. J Qual Life Res. (2022) 31:1043–56. 10.1007/s11136-021-02977-0 34463862 PMC8406389

[17] Zapata-Lamana R Poblete-Valderrama F Ledezma-Dames A Pavón-León P Leiva AM Fuentes-Alvarez MT. Health, functional ability, and environmental quality as predictors of life satisfaction in physically active older adults. Soc Sci. (2022) 11:265. 10.3390/socsci11060265

[18] Park HY Yu Seon. Transitional Analysis on the healthcare services of public health centers during the COVID-19 pandemic. Korean Publ Health Res. (2021) 47:33–44. 10.22900/kphr.2021.47.3.004

[19] Lee S-J Hong N-S Kim K-Y Ryu DH Bae SG Kim J-M. Citizen participation in the process of establishing the community health plan: based on the results of roundtable discussions to resolve the health disparity. J Korea Acad Ind Coop Soc. (2021) 22:151-161. 10.5762/KAIS.2021.22.5.151

[20] Jae-Ik Cho U-SY. Health Living Support Center following the Function Change of Public Health Center- Operation Satus and Sace Composition of the Healthy Living Support Center. J Archit Inst Korea. (2022) 42:514–7.

[21] Pendergrass J Ranganathan C. Institutional factors affecting the electronic health information exchange by ambulatory providers. Health Policy Technol. (2021) 10:100569. 10.1016/j.hlpt.2021.100569

[22] Park Y Park S Lee M. Analyzing community care research trends using text mining. J Multidiscip Healthc. (2022) 15:1493. 10.2147/JMDH.S366726 35873091 PMC9297196

[23] Park B Yi K Choi S Seo S. Choi SJ. Typology of community health vulnerabilities and their effects on health status by type-using community health survey. J Korean Acad Community Health Nurs. (2021) 32:281–91. 10.12799/jkachn.2021.32.3.281

[24] Penton H Dayson C Hulme C Young T. A qualitative investigation of older adults' conceptualization of quality of life and a think-aloud content validation of the EQ-5D-5L, SF-12v2, Warwick Edinburgh Mental Wellbeing Scale, and Office of National Statistics-4. Value Health. (2022) 25:2017–27. 10.1016/j.jval.2022.04.1735 35760713

[25] Jameel A Asif M Hussain A Hwang J Bukhari MH Mubeen S. Improving patient behavioral consent through different service quality dimensions: assessing the mediating role of patient satisfaction. Int J Environ Res Public Health. (2019) 16:4736. 10.3390/ijerph16234736 31783526 PMC6926908

